# Modulation of corticospinal excitability by transcranial magnetic stimulation in children and adolescents with autism spectrum disorder

**DOI:** 10.3389/fnhum.2014.00627

**Published:** 2014-08-13

**Authors:** Lindsay M. Oberman, Alvaro Pascual-Leone, Alexander Rotenberg

**Affiliations:** ^1^Department of Neurology, Berenson-Allen Center for Noninvasive Brain Stimulation, Beth Israel Deaconess Medical Center – Harvard Medical SchoolBoston, MA, USA; ^2^Neuromodulation Program and Division of Epilepsy and Clinical Neurophysiology, Department of Neurology, Boston Children’s Hospital – Harvard Medical SchoolBoston, MA, USA; ^3^Neuroplasticity and Autism Spectrum Disorder Program, E. P. Bradley Hospital, East Providence, RIUSA; ^4^Department of Psychiatry and Human Behavior, Warren Alpert Medical School of Brown University, East Providence, RIUSA

**Keywords:** autism spectrum disorders, transcranial magnetic stimulation, development, plasticity, GABA, theta burst stimulation

## Abstract

The developmental pathophysiology of autism spectrum disorders (ASD) is currently not fully understood. However, multiple lines of evidence suggest that the behavioral phenotype may result from dysfunctional inhibitory control over excitatory synaptic plasticity. Consistent with this claim, previous studies indicate that adults with Asperger’s Syndrome show an abnormally extended modulation of corticospinal excitability following a train of repetitive transcranial magnetic stimulation (rTMS). As ASD is a developmental disorder, the current study aimed to explore the effect of development on the duration of modulation of corticospinal excitability in children and adolescents with ASD. Additionally, as the application of rTMS to the understanding and treatment of pediatric neurological and psychiatric disorders is an emerging field, this study further sought to provide evidence for the safety and tolerability of rTMS in children and adolescents with ASD. Corticospinal excitability was measured by applying single pulses of TMS to the primary motor cortex both before and following a 40 s train of continuous theta burst stimulation. 19 high-functioning males ages 9–18 with ASD participated in this study. Results from this study reveal a positive linear relationship between age and duration of modulation of rTMS after-effects. Specifically we found that the older participants had a longer lasting response. Furthermore, though the specific protocol employed typically suppresses corticospinal excitability in adults, more than one third of our sample had a paradoxical facilitatory response to the stimulation. Results support the safety and tolerability of rTMS in pediatric clinical populations. Data also support published theories implicating aberrant plasticity and GABAergic dysfunction in this population.

## INTRODUCTION

Autism spectrum disorder (ASD) is diagnosed clinically, based on the key symptoms including qualitative impairments in social communication and the presence of restricted and repetitive behaviors (APA, 2013). However, the variability of the clinical phenotype of ASD is quite large and symptoms can manifest over a range of ages in childhood. Thus, ASD diagnosis can be challenging and is often not made until 3–5 years of age. For this reason, a physiologic ASD biomarker is highly desirable.

Several lines of evidence suggest that an impairment of GABAergic transmission may be critical in the pathophysiology of ASD (see Coghlan et al., 2012 for a review). GABA plays a key role in regulating neuronal excitability via feedback and feed-forward inhibition (Sutor and Luhmann, 1995; Petroff, 2002; Madsen et al., 2008; Huang, 2009). While in the mature brain GABA acts as an inhibitory neurotransmitter, during the embryonic and the perinatal period, GABA is excitatory (Cherubini et al., 1991). It is hypothesized that at least some forms of autism result from an imbalance between excitation and inhibition in local circuits involved in sensory, mnemonic, social, and emotional processes (Rubenstein and Merzenich, 2003; Markram and Markram, 2010). Ben-Ari et al. (2012), for instance, suggest that a dysfunction in the shift of GABA from excitation to inhibition may contribute to this imbalance.

Empirical support for the role of aberrant GABA signaling in the pathophysiology of ASD comes from both human and animal model research. A recent study conducted by Tyzio et al. (2014) found that GABA had excitatory action in two animal models of ASD [rats exposed to valproate in utero (VPA) and mice carrying the fragile X mutation (FRX)]. Furthermore, maternal pretreatment with bumetanide, forcing the shift of GABA in the offspring from excitatory to inhibitory, resulted in the restoration of typical electrophysiological and behavioral phenotypes in affected animals (Tyzio et al., 2014).

Human studies have found reduced GABA receptor expression (Fatemi et al., 2009a,b, 2010) as well as a 50% reduction in enzymes that synthesize GABA [*glutamic acid decarboxylase* (GAD) 65 and 67; Fatemi et al., 2002; Yip et al., 2007] in individuals with ASD. Furthermore, a recent study (Gaetz et al., 2014) identified significant reduction in the GABA MRS signal in the motor cortex of patients with ASD, and a marginally significant (*p* = 0.054) positive correlation between the GABA signal and age. Among the many roles of GABAergic circuits during development, one is lateral inhibition across neighboring minicolumns in the cortex. Consistent with an impairment in GABAergic transmission, postmortem studies have found a reduction in the horizontal spacing between minicolumns (Casanova et al., 2002). These abnormalities in the GABA system may directly contribute to altered anatomical and functional connectivity, and suggest a mechanism underlying the neurological and behavioral phenotype of ASD (Blatt, 2011).

In addition to and perhaps as a consequence of excitation/inhibition imbalance, recent studies in both human and animal models implicate synaptic plasticity mechanisms in the pathophysiology of ASD (see Oberman et al., in press). While most synaptic plasticity data in ASD are derived from *in vitro* rodent brain slice models, direct measures of circuit level plasticity in humans can be obtained by transcranial magnetic stimulation (TMS) paradigms (Ziemann, 2004; Huang et al., 2005; Thickbroom, 2007; Huerta and Volpe, 2009). In TMS, the cortex is stimulated focally by small intracranial electrical currents that are generated by a powerful and fluctuating extracranial magnetic field (Barker et al., 1985; Kobayashi and Pascual-Leone, 2003; Hallett, 2007). A number of experimental TMS measures of brain plasticity have been introduced, and provide the only noninvasive capacity to measure human phenomena that closely resemble long-term potentiation (LTP) and long-term depression (LTD). TMS is safe and well-tolerated, even in pediatric populations, if appropriately guidelines and recommendations are followed (Garvey and Gilbert, 2004; Rajapakse and Kirton, 2013).

Single-pulse TMS combined with EMG, EEG, fMRI, or other brain imaging methods can be used to quantify cortical reactivity before and following a given intervention (Pascual-Leone et al., 2011) providing an index of brain plasticity in response to said intervention. Recently, patterned bursting protocols have been developed that mimic paradigms used to assess synaptic plasticity in animal models (Huang et al., 2005, 2008). Specifically, theta burst stimulation (TBS) involves application of three bursts of 50-Hz rTMS repeated every 200 ms either continuously for a total of 40 s or intermittently (every 8 s) for about 3 min. When applied to the motor cortex, continuous TBS (cTBS) and intermittent TBS (iTBS) result in depression and potentiation of cortical reactivity as indexed through suppression and facilitation of motor evoked potentials (MEPs), respectively (Huang et al., 2005). Results from animal and human studies indicate that TBS modulatory effects on cortical reactivity reflect synaptic plasticity mechanisms (Cardenas-Morales et al., 2011). Specifically, and relevant to the present experiment, published data suggest that cTBS leads to enhancement of GABAergic inhibition (Stagg et al., 2009; Benali et al., 2011).

Notably, compared to other rTMS protocols, TBS has the advantage of lower stimulation intensities and shorter durations than conventional protocols making this protocol more suitable for use in clinical and pediatric populations. The safety and tolerability of this protocol has recently been evaluated and shown to be safe in healthy children and in children with Tourette Syndrome (Wu et al., 2012).

In a recent study (Oberman et al., 2012) we used the cTBS paradigm in 20 adults with Asperger’s syndrome (high functioning ASD), and found them to show greater and longer-lasting suppression of cortical reactivity in the motor cortex following cTBS as compared to age-, gender-, and IQ-matched controls. The latency to return to baseline following TBS was on average between 80 and 90 min in the ASD group compared to 25–30 min in the controls. This finding was confirmed in a separate cohort of 15 individuals (Oberman et al., 2012). Interestingly, and consistent with other studies, there was no significant group difference in measures of basic excitability as assessed by resting and active motor threshold (Theoret et al., 2005; Oberman et al., 2012; Enticott et al., 2013) or response to single pulse TMS (Oberman et al., 2012). Thus, the excessive modulation of excitability in response to stimulation (a putative measure of plasticity) is not primarily attributable to differences in baseline excitability.

In the current study, we extended our age range to include data from 19 children and adolescents with high-functioning ASD (HF ASD) to explore the effect of development on the response to the cTBS paradigm. As the application of rTMS to the understanding and treatment of pediatric neurological and psychiatric disorders is an emerging field (Frye et al., 2008; Croarkin et al., 2011), this study additionally aimed to provide evidence for the safety and tolerability of TBS in HF children and adolescents with ASD.

## MATERIALS AND METHODS

### PARTICIPANTS

We studied 19 males with HF ASD, age 9–18 years (See **Table 1** for demographic characteristics of the sample). All participants gave informed consent or assent, which was also obtained from a parent or guardian to participate in the study. The study was reviewed and approved by the institutional review board at Boston Children’s Hospital. Participants were recruited through local community advertisement. All participants had IQ > 80 based on the Weschler Abbreviated Scale of Intelligence (WASI). All met DSM-IV-TR criteria for Autism, Asperger’s Syndrome or PDD-NOS, and met criteria for ASD on the Autism Diagnostic Observation Schedule, Module 4 (ADOS). Some participants also had comorbid symptoms including inattention, anxiety, irritability, and obsessive-compulsive behaviors (See **Table 1**). All participants were given a comprehensive neurological exam by a board-certified pediatric neurologist (Alexander Rotenberg) to confirm normal gross motor and fine motor function. Lastly, all participants were screened following published recommendations (Rossi et al., 2009) to ensure that they did not have any condition that would put them at greater risk of an adverse event related to TMS (e.g., a personal or immediate family history of epilepsy).

**Table 1:** Sample characteristics.

Participant number	Age	IQ	ADOS Score	Comorbid symptoms	Neuroactive medications	Response to cTBS
1	11	100	13	Anxiety	Citalopram	Suppression
2	9	94	7	None	None	Facilitation
3	12	86	10	Anxiety, Inattention	Citalopram, atomoxetine	Suppression
4	9	87	9	Anxiety ADHD	None	Suppression
5	14	115	8	Anxiety, ADHD	Citalopram, atomoxetine	Facilitation
6	14	99	9	None	None	Facilitation
7	10	106	12	None	None	Suppression
8	9	88	7	None	None	Facilitation
9	10	89	10	ADHD	Buspirone	Suppression
10	11	93	9	Obsessive-Compulsive Behaviors, Anxiety	Sertraline, citalopram	Facilitation
11	11	115	7	ADHD	Methylphenidate	Suppression
12	13	129	9	Inattention	Atomoxetine	Suppression
13	14	115	8	ADHD	Atomoxetine	Suppression
14	10	102	7	Irritability	Risperidone	Suppression
15	11	103	14	Inattention	Guanfacine, methylphenidate	Facilitation
16	13	102	7	ADHD	Methylphenidate	Facilitation
17	18	121	12	None	None	Suppression
18	18	83	13	None	None	Suppression
19	17	81	13	None	None	Suppression
**AVERAGE**	**12.26**	**100.42**	**9.58**			

### STIMULATION AND RECORDING

To evaluate modulation of corticospinal excitability (a putative index of cortical plasticity mechanisms) and specifically GABAergic inhibition, cTBS was applied to the primary motor cortex. The cTBS paradigm used in the current study was identical to that described by Huang et al. (2005) and applied in previous studies in our laboratory (Oberman et al., 2010, 2012). The protocol consisted of three pulses of 50 Hz stimulation repeated at 200-ms intervals for 40 s (for a total of 600 pulses) at an intensity of 80% of active motor threshold (AMT). Corticospinal excitability was assessed prior to and following cTBS by measuring peak-to-peak amplitude of MEPs induced in the contralateral first dorsal interosseus (FDI) muscle in response to single-pulse TMS. These single pulses were applied at a rate of approximately 0.1 Hz (a random jitter of ±1 s was introduced to avoid any train effects). Three batches of 10 MEPs were recorded prior to cTBS and used as a baseline. Beginning at 5 min following cTBS, batches of 10 MEPs were measured at periodic intervals (5, 10, 15, 20, 30, 40, 50, 60, 70, 80, 90, 105, and 120 min) until the MEPs returned to baseline levels to track changes in MEP amplitude over time. The participant was asked to remain relaxed during the entire study. Muscle activity was monitored throughout the session with EMG surface electrodes. TMS was only applied when the EMG signal indicated that the participant’s FDI muscle was in a relaxed state. Any trials where the participant voluntarily contracted the muscle within 1000 ms of the TMS pulse were not included in the analysis.

To measure TMS induced MEPs, EMG surface electrodes were placed in a belly tendon montage over the FDI muscle of participants’ right hands. Raw signals were amplified and bandpass-filtered between 20 and 2000 Hz. EMG signals were sampled at a rate of 5000 Hz. All stimulation (single-pulse TMS and TBS) was delivered using a hand-held 70 mm figure-of-eight coil attached to a Magstim Super Rapid stimulator (The MagStim Company Ltd., Whitland, UK). The coil was placed tangentially to the scalp with the handle pointing posteriorly. All stimulation was applied over the hand area of the left motor cortex and individually localized for each participant based on the optimal position for eliciting MEPs in the right FDI. The stimulation intensity for baseline and post-TBS single pulses was set at 120% of each individual’s resting motor threshold (RMT) while the TBS itself was delivered at 80% of AMT. RMT and AMT were defined following recommendation from the International Federation of Clinical Neurophysiology. RMT was defined as the minimum single-pulse TMS intensity required to induce an MEP in the contralateral FDI of >50 μV peak-to-peak amplitude on more than five out of ten consecutive trials while the target muscle was at rest. AMT was defined as the minimum single-pulse TMS intensity required to induce an MEP in the contralateral FDI of >200 μV peak-to-peak amplitude on more than five out of ten consecutive trials while the target muscle was held at approximately 20% of the maximal contraction. To precisely target the stimulation site (primary motor cortex) and keep the brain target constant throughout the stimulation session, we used a frameless stereotactic neuronavigation system (Brainsight, Rogue Research Inc., Montreal, QC, Canada).

### DATA ANALYSIS

Data were analyzed using MatLab version 8.1 and SPSS version 22. Data analysis followed the methods described and applied in previous studies in our laboratory (Oberman et al., 2010, 2012). Average MEP amplitude values were calculated at baseline prior to TBS and starting five minutes after TBS and continuing until the average amplitude returned to within the 95% confidence interval of the baseline amplitude and did not return to outside that interval on subsequent time-point measures. MEP amplitudes were standardized, forming a ratio of MEP amplitudes following TBS relative to average baseline MEP amplitude for each individual.

Cubic spline interpolation was used to create smooth curves through the data points. Spline interpolation is a piecewise continuous function defined by third-degree polynomials in the intervals of a limited range of known data points (in this case, the time-points at which MEP data were collected with batches of 10 single TMS pulses). The use of spline interpolation on TMS data has been validated (Borghetti et al., 2008) and used in previous studies to evaluate degree and duration of modulation of MEP amplitudes following cTBS (Freitas et al., 2011). As an index of the duration of the TBS-induced modulation of cortico-spinal excitability, we defined, for each participant, the time-point (“time to baseline”) at which post-cTBS MEP amplitude returned to the average MEP amplitude at baseline, i.e., the time-point at which the spline crossed the MEP threshold.

A natural log transformation was applied to the data prior to analysis as tests of normality indicated that the data was significantly different than normal. A Pearson product-moment correlation coefficient was calculated to assess the degree of relationship between age and duration of response to cTBS.

### SIDE EFFECT MONITORING

Immediately following the TMS session a side effects questionnaire was completed by the experimenter. Participants were asked to report whether they experienced any of the following side effects: headache, neck pain, scalp pain or irritation, difficulty hearing, thinking or concentrating, change in mood, or any other change or side effect they experienced. The experimenter also noted whether the participant experienced a syncopal event or seizure. The participant also received a call the day after the TMS session and was once again asked to report whether they experienced any of the above side effects or to report any other side effect they experienced after they left the hospital. If the participant reported any side effect either immediately following the stimulation or the following day, its severity and duration were documented.

## RESULTS

### TMS SAFETY AND TOLERABILITY

All participants tolerated TBS and single-pulse stimulation without serious adverse event. One participant had a mild headache after stimulation that was alleviated with a single acetaminophen dose. Two participants had mild fatigue after the session that resolved the following day. No other adverse events were reported.

### AGE-DEPENDENT RESPONSE TO cTBS

**FIGURE 1:**
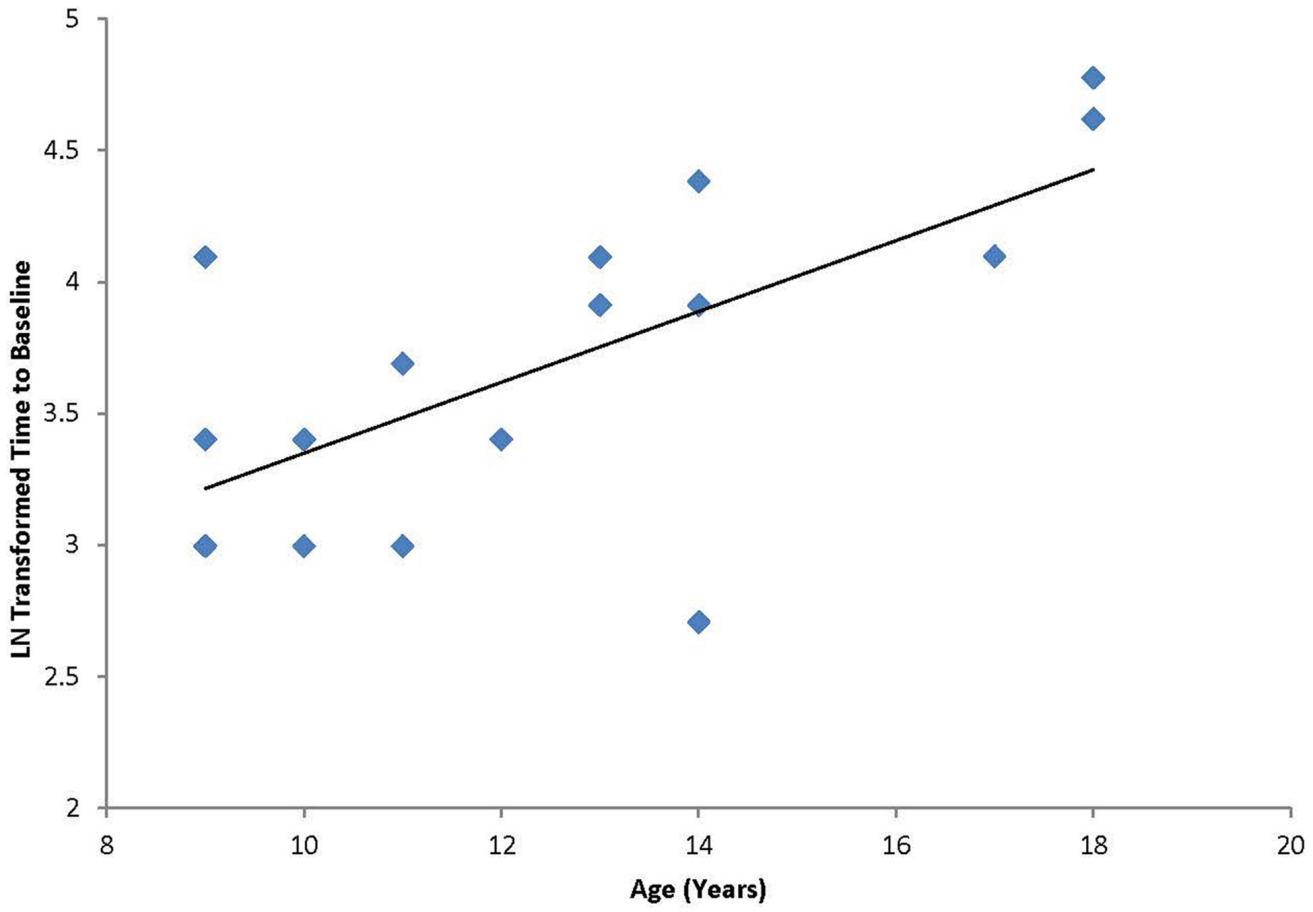
**Correlation between age and the natural log transformed “time to baseline” for each individual following cTBS.** Graph shows a significant positive relationship between duration of modulation and age.

A Pearson product-moment correlation coefficient was calculated to test the hypothesis that a linear relationship existed between age and duration of response to cTBS. As described above, response to the cTBS protocol was defined as duration of effect as defined by the number of minutes following cTBS before the participant returned to baseline excitability levels (“time-to-baseline”; *M* = 46.3 min, SD = 29.3 min). The results of the analysis indicated that there was a significant positive linear relationship between age and duration of response [*r*(17) = 0.660, *p* < 0.01; **Figure 1**].

**FIGURE 2:**
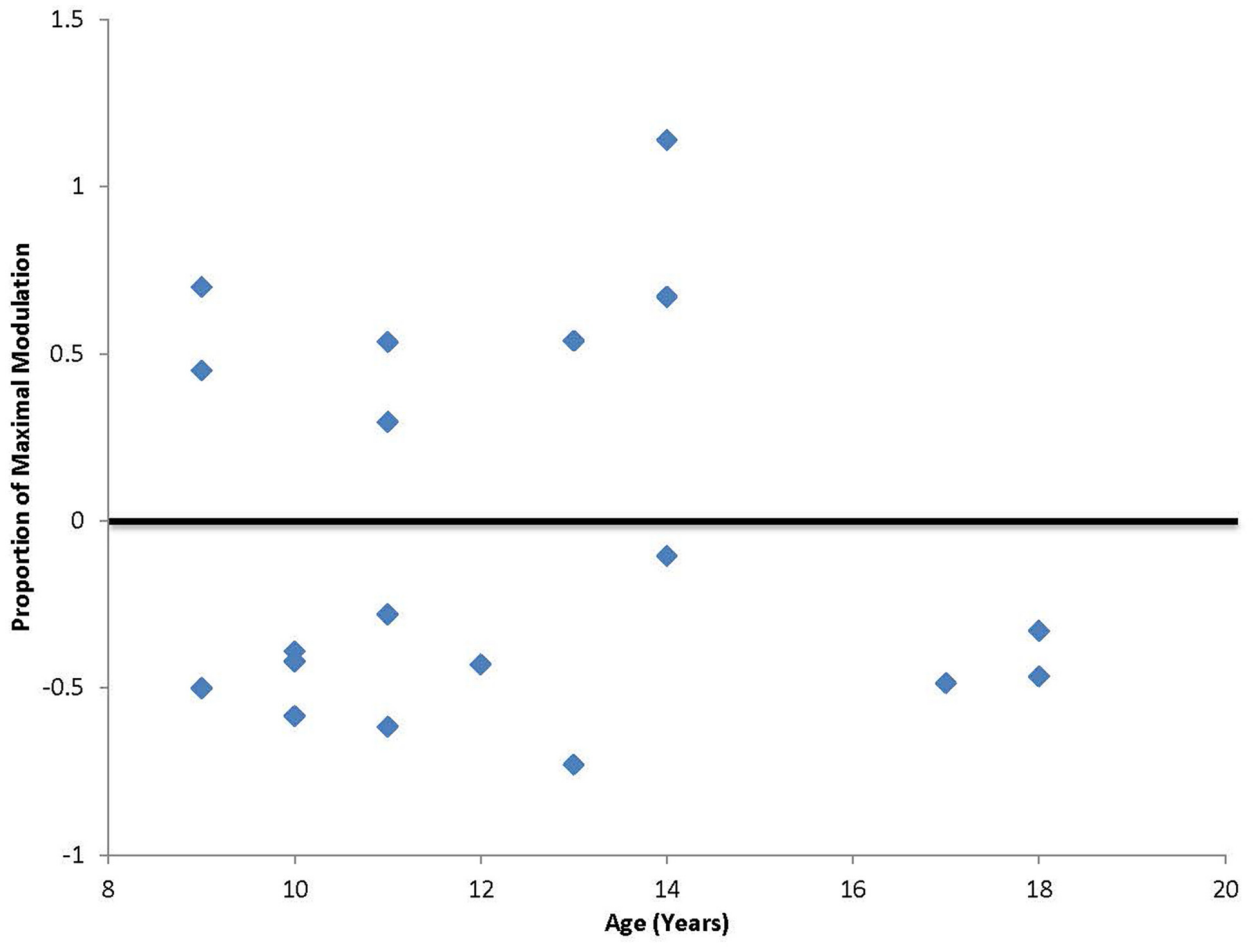
**Degree of maximal suppression or facilitation following cTBS.** Values falling above zero indicate paradoxical facilitation in response to the typically suppressive cTBS protocol. Seven out of 20 participants in this study, ages 9, 11, 13, and 14 showed this paradoxical facilitation.

In addition to the planned analyses, it was noted that unlike our previous study that included only adult participants, a third of the participants (7 out of 19; ages 9, 11, 13, and 14) had a paradoxical facilitation in response to the cTBS protocol (**Figure 2**). Additional analyses were conducted excluding the seven participants who facilitated and the significant positive correlation between age and duration of response remained [*r*(10) = 0.62, *p* < 0.05] with a Mean duration of 49.2 min (SD = 33.8 min). The subgroup of participants who displayed the paradoxical facilitation response was not predicted by comorbid symptoms or medication (χ^2^ = 0.091, *p* = 0.76).

## DISCUSSION

As the application of rTMS protocols to children increases, it is critical to evaluate the safety and tolerability of this procedure in these vulnerable populations. In the current study, all participants tolerated the stimulation and reported only minor discomforts that resolved quickly following the procedures. These findings add to the literature suggesting that rTMS is safe and well tolerated in children and in individuals with ASD (Garvey and Gilbert, 2004; Frye et al., 2008; Croarkin et al., 2011; Wu et al., 2012; Oberman et al., 2013; Rajapakse and Kirton, 2013). Systematic monitoring and documentation of side effects is critical moving forward to ensure that both participants and investigators have an accurate sense of both the range and frequency of side effects of rTMS in clinical and pediatric populations.

Our findings are also the first step toward the study of the developmental regulation of the cTBS effect and reveal a positive linear relationship between age and duration of modulation of cTBS. Specifically we found that the older participants had a longer lasting response. On the surface this may appear counter intuitive if we consider that response to TBS has been used by our group and others to indicate degree of plasticity. One would imagine that younger children have a greater, not lesser, capacity for plasticity (Huttenlocher, 2002). However, cTBS is thought to model LTD-like plasticity (Huang et al., 2008) and is related to GABAergic inhibitory tone. Thus, perhaps LTD-like plasticity or GABAergic inhibition increases over development, especially during adolescence (Selemon, 2013). As we did not perform iTBS, we cannot speak to the development of LTP-like plasticity in this sample, however, it would be important to evaluate this process as well. Additionally, the current study did not include a sham control condition or any other rTMS protocol, thus it is unclear whether the results are specific to the cTBS paradigm.

Recently, the molecular mechanisms underlying the changes in cortical excitability induced by cTBS have been studied using MRS (Stagg et al., 2009). The findings reveal that the effects of cTBS are mediated by changes in the local activity of inhibitory interneuronal cortical pathways (as measured by changes in cortical GABA concentration in the primary sensorimotor cortex; Stagg et al., 2009). Consistent with the idea that younger children have less inhibitory tone, studies using paired pulse measures of intracortical inhibition have found that children display decreased levels of suppression as compared to adolescents or adults (Walther et al., 2009). This study further claimed that reduced GABA mediated intracortical inhibition may facilitate excitatory (LTP-like) cortical plasticity and motor learning in children. Thus, the current results, although obtained from individuals with ASD, provide further evidence of increasing capacity for LTD-like suppression of cortical excitability across childhood.

Additionally, the finding that over one third of our sample had a paradoxical facilitatory response to cTBS supports the notion of GABAergic dysfunction in ASD. During typical development, GABA currents shift from excitatory to inhibitory through a maturation of chloride transport mechanisms and an age-dependent reduction of intracellular chloride concentration [(Cl^-^_i_; Ben-Ari et al., 2007)]. However, a recent study finds that two ASD animal models (rodent valproate and fragile X models) show excitatory GABA activity well beyond the age where wild-type animals’ GABA activity has shifted to inhibition (Tyzio et al., 2014). In these animals administration of a GABA agonist (isoguvacine) led to an *increase* in spike frequency in neurons recorded from hippocampal slices as compared to a decrease in wild-type animals. Additionally, the in utero administration of bumetanide, a chloride importer antagonist that reduces intracellular chloride accumulation thereby promoting the shift of GABA from excitation to inhibition, resulted in the restoration of typical electrophysiological and behavioral phenotypes in affected offspring (Tyzio et al., 2014). These preclinical data support the hypothesis that a dysfunction in this shift may contribute to the pathophysiology of ASD (Ben-Ari et al., 2012). Ben-Ari and colleagues have proposed that this dysfunction may be a result of increased intracellular ([Cl^-^]_i_) concentrations in individuals with ASD. This is further supported by a study reporting paradoxical *increases* in hyperactivity in six out of seven and aggression in seven out of seven children with ASD who were treated with diazepam (Marrosu et al., 1987). Furthermore, in a recent clinical trial where bumetanide, was given to children with ASD results showed improvement in ASD symptoms as measured by the Childhood Autism Rating Scale (CARS) and the Repetitive and Restricted Behavior Scale (RRB) as well as a reduction in aberrant behavior as measured by the Aberrant Behavior Checklist (ABC; Lemonnier and Ben-Ari, 2010). Our findings suggest that one could do an analogous study in humans to explore whether bumetanide would normalize the cTBS modulation in those with a paradoxical facilitation and if this normalization corresponded to improved behavioral symptoms.

We recently suggested that the neurological and behavioral ASD phenotypes are associated with altered brain plasticity that can be measured noninvasively by TMS (Oberman et al., in press). As our data showing age-dependence of the cTBS response suggest, the timing of plastic brain changes may be important for optimal development of cortical circuitry. As ASD is a developmental disorder it would be critical to evaluate the developmental trajectory of abnormalities in a putative mechanism underlying the phenotype.

As the current study did not include healthy control participants or females, it is not clear whether the observed developmental trajectory shown by these males with ASD is similar to what may be obtained in neurotypical individuals or females with ASD. It is possible that variables such as head size or myelination could have led to the observed correlation with age. However, as reviewed above, multiple lines of evidence point toward aberrant GABAergic transmission in ASD. Thus, it will be important to evaluate these measures in a healthy developing population and female ASD population to compare the typical developmental trajectory to that shown in males with ASD. Additionally, follow-up translational studies, analogous to what has been done in animal models, directly testing the relationship between GABA receptor expression [measured by [^11^C] FMZ PET (Maziere et al., 1984)] or concentration [measured by MRS (Mescher et al., 1998)] and measures of cortical reactivity in humans with ASD are needed.

In the current study, we focused on primary motor cortex in the left hemisphere. Thus, it is unclear whether other cortical regions would show similar developmental trajectories or whether there would be a laterality effect in these individuals. The left primary motor cortex was chosen in this study for two reasons. First, MEPs are the standard index used to quantify the effect of TBS protocols. Other indices of cortical excitability outside the motor cortex (e.g., based on electroencephalographic measures) have not yet been well validated for this application. We chose the left hemisphere as it is typically the dominant hemisphere for both right- and left-handed individuals. Second, although motor abnormalities are not considered core symptoms of ASD, many studies have reported motor deficits in individuals with ASD, including alterations in motor milestone development (Teitelbaum et al., 1998), clumsiness, motor incoordination, disturbances in reach-to-grasp movement (Miyahara et al., 1997; Ghaziuddin and Butler, 1998; Mari et al., 2003), deficits in gross and fine motor movement (Noterdaeme et al., 2002), and impaired postural control (Kohen-Raz et al., 1992; Minshew et al., 2004). It has also been suggested that these motor deficits may underlie the core deficits in ASD (Mostofsky and Ewen, 2011).

Our results support the safety and tolerability of TBS in the pediatric ASD populations. As we continue to enhance our understanding of the relationship between the response to cTBS with GABAergic inhibition and GABAergic dysfunction with ASD pathophysiology we suggest that cTBS may be a practical biomarker of GABAergic dysfunction in this population.

## AUTHOR CONTRIBUTIONS

Lindsay M. Oberman designed the study, collected the data, analyzed the data, and wrote the manuscript. Alvaro Pascual-Leone contributed to the study design, interpretation of the data, and the writing of the manuscript, Alexander Rotenberg contributed to the study design, interpretation of the data, supervised and contributed to data collection, and contributed to writing of the manuscript.

## Conflict of Interest Statement

Alvaro Pascual-Leone serves on the scientific advisory boards for Nexstim, Neuronix, Starlab Neuroscience, Neuroelectrics, and Neosync; and is listed as an inventor on several issued and pending patents on the real-time integration of transcranial magnetic stimulation (TMS) with electroencephalography (EEG) and magnetic resonance imaging (MRI). Alexander Rotenberg is listed as an inventor on a patent for apparatus and method of use of TMS in epilepsy. He is a co-founder of Neuromotion Inc.
